# Case Report: New Application of a Gufoni Maneuver Variation for Apogeotropic Lateral Semicircular Canal Benign Paroxysmal Positional Vertigo

**DOI:** 10.3389/fneur.2022.902758

**Published:** 2022-06-10

**Authors:** Jiaoxuan Dong, Ling Li, Songbin He, Haipeng Liu, Fangyu Dai

**Affiliations:** ^1^Department of Neurology, Zhoushan Hospital, Wenzhou Medical University, Zhoushan, China; ^2^Research Centre for Intelligent Healthcare, Coventry University, Coventry, United Kingdom

**Keywords:** canalith repositioning procedure, benign paroxysmal positional vertigo (BPPV), semicircular canals, Gufoni maneuver, case report, apogeotropic variant

## Abstract

**Background:**

Several canalith repositioning procedures (CRPs) such as Gufoni maneuver have been proposed to treat the apogeotropic lateral semicircular canal variant of BPPV (LC-BPPV). The reported success rate varied widely in different studies. Research showed that there was a risk of treatment failure due to insufficient repositioning of the debris. So far, there is insufficient evidence to recommend a preferable CRP for apogeotropic LC-BPPV.

**Case description:**

A 49-year-old woman and a 48-year-old man diagnosed with apogeotropic LC-BPPV relapse were treated with original Gufoni maneuver for apogeotropic variant but no satisfactory result was obtained. A variation of Gufoni maneuver originally proposed for the geotropic variant was applied to detach otoconia toward the utricle or the non-ampullary arm. Apogeotropic nystagmus was successfully transformed into the geotropic variant. The subsequent Gufoni maneuver was successful. On a 64-year-old male with untreated apogeotropic LC-BPPV, we performed the Gufoni maneuver variation and observed a change in nystagmus direction. In all the three cases, no relapse of vertigo was reported after 1 month.

**Conclusion:**

The new application of Gufoni maneuver variation may improve the treatment of apogeotropic LC-BPPV. Treatment efficacy and patient-specific optimization such as head rotation angle deserve a large-scale validation and further investigation.

## Introduction

Benign paroxysmal positional vertigo (BPPV) is the most frequent peripheral vestibular disorder induced by changes in head position relative to the gravitational axis (1). Of all BPPV cases, LC-BPPV is the second commonest subtype after posterior semicircular canal BPPV (PC-BPPV) (2). The apogeotropic variant of LC-BPPV is attributed to otoliths attached to the cupula (cupulolithiasis) on the canal side (Cup-C) or the utricular side (Cup-U), or free-floating otoliths in the ampullary arm of the LC (3). Owing to the three different pathological types, the treatment of this variant is difficult. Several canalith repositioning procedures (CRPs) have been proposed for the apogeotropic variant (4), such as a new variation of Gufoni maneuver, i.e., the Zuma maneuver (5, 6), and recently, the mastoid vibration maneuver (7).

As one of the commonest CRP for LC-BPPV, the Gufoni maneuver has two variations. The original variation proposed for the geotropic variant of LC-BPPV consists of a quick movement onto the unaffected side followed by a downward head rotation, i.e., the “Gufoni nose-down” variation (8). Ciniglio Appiani et al. described the details of this variation in a publication in English in 2001 (9). In contrast, the variation originally proposed for apogeotropic LC-BPPV consists of a quick movement onto the affected side followed by an upward head rotation, i.e., the “Gufoni nose-up” variation (10).

The Gufoni maneuver for the apogeotropic variant (Gufoni nose-up variation) was considered efficient in converting LC-BPPV from the apogeotropic to the geotropic form (10). A randomized clinical trial indicated that the Gufoni maneuver was more effective than the head-shaking maneuver, with a 73.1% success rate (11). However, the reported success rate varied widely in different studies, which could be considerably lower. Research showed that there was a risk of treatment failure due to insufficient repositioning of the debris when the Gufoni nose-up variation was applied in apogeotropic LC-BPPV (12). So far, there is insufficient evidence to recommend a preferable CRP for apogeotropic LC-BPPV (13).

Recently, we had observed three patients experiencing severe apogeotropic LC-BPPV. We first performed the Gufoni nose-up variation but failed to convert it into the geotropic variant. Thus, we adopted the Gufoni maneuver for the geotropic variant (Gufoni nose-down variation) to treat the apogeotropic variant and achieved good efficiency.

## Methods

**Figure 1:**
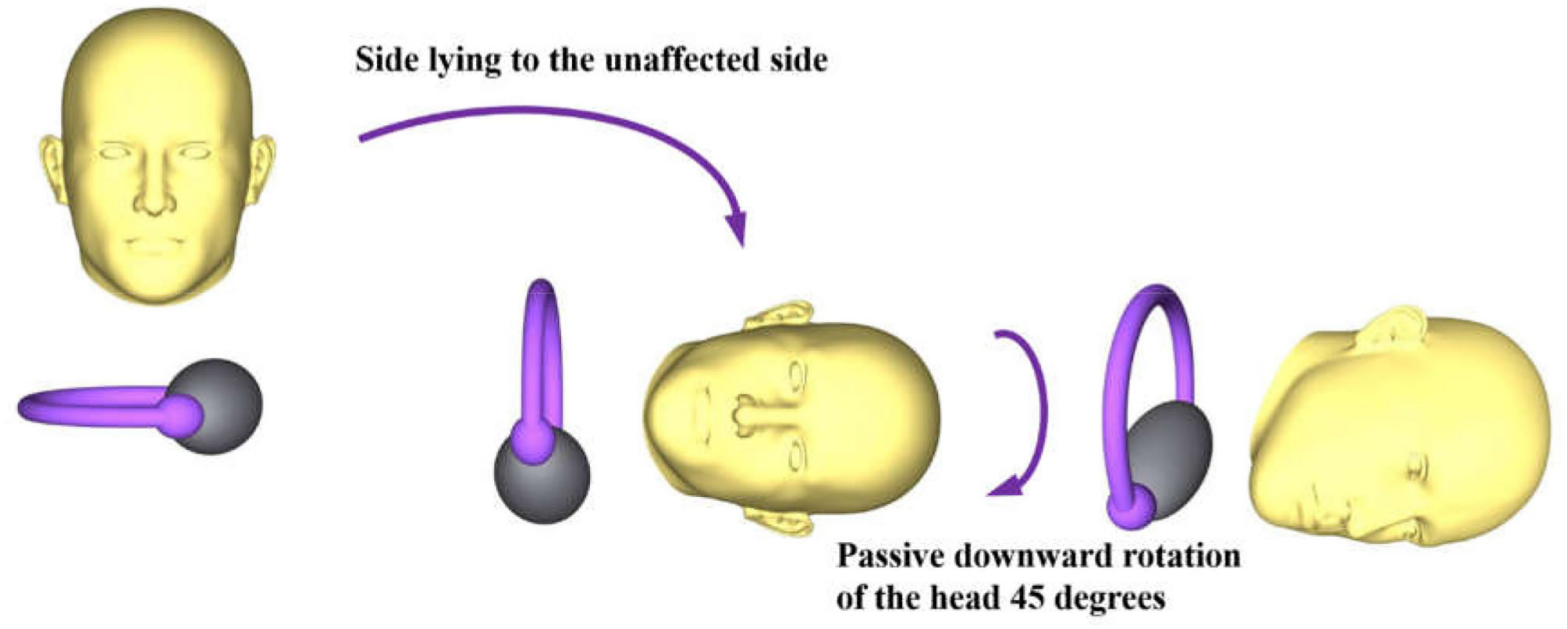
New application of the Gufoni maneuver for apogeotropic LC-BPPV. The right side is the affected side.

In the Gufoni nose-up variation for the apogeotropic variant, the patient is quickly moved from the sitting position onto the affected side, followed by a rapid 45° upward head rotation before returning to the sitting position (14). In contrast, the Gufoni nose-down variation adopted in this study, which is based on the Gufoni maneuver presented by Ramos et al. (14), starts from the sitting position, from which the patient is moved quickly onto the unaffected side, followed by a quick 45° downward turn and holding for 2 min (Figure 1). If the transformation of the nystagmus direction is achieved, the Gufoni maneuver for the geotropic variant (Gufoni nose-down variation) will be further performed.

## Case Presentation

### Case 1

A 49-year-old female with no history of vertigo experienced her first vertigo attack 3 months ago without any symptoms of headache, tinnitus, or deafness. The vertigo episode was related to her head position with a short duration of ~1–2 min. The patient had no previous history of other chronic diseases. She had been diagnosed with apogeotropic LC-BPPV in our neurology department and was repositioned with the Gufoni maneuver (Gufoni nose-up variation). The vertigo relapsed 1 day ago before her second admission. The neurophysical examination showed negative results. No spontaneous or gaze-evoked nystagmus was found. The Brain CT showed no structural lesions. The supine roll test was performed with the patient lying on the examination table. Her head was briskly rotated to the right side from the supine position. An apogeotropic nystagmus lasting over 1 min was observed. After the maximal head yaw movement to the right, the patient's head was brought to the supine position and then briskly rotated to her left, which elicited a stronger apogeotropic nystagmus lasting over 1 min. According to Ewald's second law, the right side was the affected side. We performed the Gufoni nose-up variation once a day for 1 week, but there was no transformation of nystagmus direction. Then, we performed the Gufoni nose-down variation. The patient was brought onto the left side from sitting position. A horizontal nystagmus beating toward the uppermost ear was evoked. After a few seconds, the patient's head was inclined 45° downward in the yaw plane and held for 2 min. During this, we observed the nystagmus transforming into the geotropic variant. Subsequent treatment with the Gufoni maneuver (Gufoni nose-down variation) was successful. At the 4-week follow-up, the patient reported no vertigo relapse.

### Case 2

A 48-year-old male with no history of vertigo suffered from serious vertigo 1 month ago and remitted spontaneously, and had a relapse 2 days ago while getting up. The vertigo attack was associated with positional change of the head. The neurological examination revealed normal cranial nerve. Strength was grade 5/5 in all four limbs with normal deep tendon reflexes. No spontaneous or gaze-evoked nystagmus was found. Brain MRI scanning revealed no structural lesions. He was diagnosed with right apogeotropic LC-BPPV in the supine roll test by observing the intensity of the nystagmus as in case 1. The Gufoni nose-up variation was performed, but no satisfactory result was obtained. Then, we performed the Gufoni nose-down variation. When the patient was briskly turned to left side-lying position from the sitting position, an apogeotropic nystagmus was observed. After a 45-degree downward turn and 2 min holding, the nystagmus was transformed into the geotropic form. He was treated with the Gufoni maneuver (Gufoni nose-down variation) subsequently and reported no relapse after 1 month.

### Case 3

A 64-year-old male had positional vertigo 3 days ago. He had no history of vertigo before. No positive result was found in brain MRI and neurophysical examination. He was diagnosed with left apogeotropic LC-BPPV in the supine roll test by observing the intensity of the nystagmus as in cases 1 and 2 (Supplementary Video 1). We performed the Gufoni nose-down variation. The patient's head was first taken to the right lateral recumbent position where an apogeotropic nystagmus was induced and subsequently quickly rotated 45° downward. In about a minute, we observed the translation of nystagmus direction. Then, he was treated with the Gufoni maneuver (Gufoni nose-down variation), and the nystagmus disappeared. He reported no vertigo relapse at 1 month follow-up.

## Discussion

**Figure 2:**
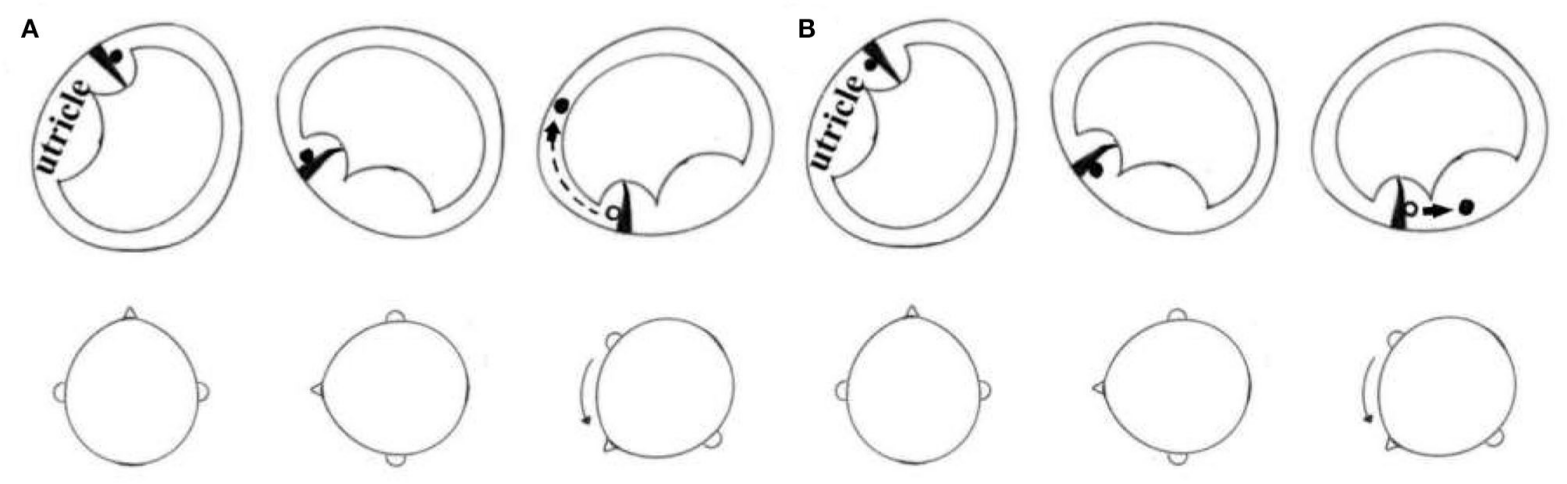
New application of the Gufoni nose-down variation. **(A)** Debris on the canal side. **(B)** Debris on the utricular side. The right side is the affected side in both cases.

An apogeotropic nystagmus that never becomes geotropic is a sign of cupulolithiasis in peripheral vertigo (14). We assume two application scenarios of the proposed maneuver (Figures 2A,B). In the first situation, the otoconia are located on the canal side (Figure 2A). In our new application of the Gufoni maneuver (i.e., using Gufoni nose-down variation for apogeotropic LC-BPPV cases), as the three patients lied on the unaffected side from the sitting position, the lateral semicircular canal was on the earth-vertical plane. In this position, the otoconia are located above the cupula. Then, the head was suddenly rotated toward the ground. These two steps required rapid angular acceleration to detach the otoconia from the cupula. If separation had been achieved in the first step, the 45-degree downward turn could facilitate the movement of the detached otoconia toward the utricle or the non-ampullary arm. During our repositioning procedures, the nystagmus was transformed to the geotropic variant. The most likely explanation was that the otoconia moved to the non-ampullary arm. We considered that the most possible diagnosis of these patients was Cup-C. In the latter form (Figure 2B), the patient will be moved from the sitting position to the side-lying position on the healthy side, attaining the most gravity-dependent position after a brisk deceleration. Under the dual effects of gravity and inertia, the otoconia will be separated from the cupula. In the second step, which requires quick deceleration, the inertia favors the otoconial movement toward utricle. There is no need for another Gufoni maneuver (Gufoni nose-down variation) if repositioning is obtained.

According to the latest guidelines, there are two ways of Gufoni maneuver for treatment of two cupulolithiasis variants (13). However, the operator could not decide the proper maneuver at the first stage of treatment. This may cause clinical treatment failure. Inspired by existing studies, we adopted the Gufoni nose-down variation to separate otoconial debris from the cupula for apogeotropic LC-BPPV. Theoretically, the two subtypes of cupulolithiasis can be treated with this repositioning maneuver. This method might provide a reference for the development of a unified repositioning maneuver for both subtypes of cupololithasis using gravity and inertia (14). We believe that a single repositioning maneuver for all the variants of LC-BPPV could help the neurotological clinical practice.

However, there are some limitations in this study. We did not perform the bow and lean test and the seated supine positioning test (SSPT), which can fully confirm the diagnosis of LC-BPPV (6). The treatment efficacy requires further large-scale validation. Further investigation is needed regarding its patient-specific optimization such as head rotation angle. Video-Frenzel goggles are also needed to remove visual fixation.

## Data Availability Statement

The original contributions presented in the study are included in the article/Supplementary Material, further inquiries can be directed to the corresponding authors.

## Ethics Statement

The studies involving human participants were reviewed and approved by Clinical Research Ethics Committee of Zhoushan Hospital. The patients/participants provided their written informed consent to participate in this study. Written informed consent was obtained from the individual(s) for the publication of any potentially identifiable images or data included in this article.

## Author Contributions

JD and FD: conception and study design. JD, HL, and FD: acquisition, analysis, and interpretation of data. JD: first draft of the manuscript. JD and HL: critical reading and manuscript revision. All the authors read and approved the submitted version.

## Funding

This study was supported by the Medical Health Science and Technology Project of Zhejiang Provincial Health Commission and the Planned Projects of Bureau of Science and Technology of Zhoushan (2020ZH065 and 2020C31048).

## Conflict of Interest

The authors declare that the research was conducted in the absence of any commercial or financial relationships that could be construed as a potential conflict of interest.

## Publisher's Note

All claims expressed in this article are solely those of the authors and do not necessarily represent those of their affiliated organizations, or those of the publisher, the editors and the reviewers. Any product that may be evaluated in this article, or claim that may be made by its manufacturer, is not guaranteed or endorsed by the publisher.
